# Shared Molecular and Functional Frameworks among Five Complex Human Disorders: A Comparative Study on Interactomes Linked to Susceptibility Genes

**DOI:** 10.1371/journal.pone.0018660

**Published:** 2011-04-21

**Authors:** Ramesh Menon, Cinthia Farina

**Affiliations:** 1 Neuroimmunology and Neuromuscular Disorders Unit, Foundation IRCCS Neurological Institute Carlo Besta, Milan, Italy; 2 Institute of Experimental Neurology (INSpe), San Raffaele Scientific Institute, Milan, Italy; University of Hawaii Manoa, United States of America

## Abstract

**Background:**

Genome-wide association studies (gwas) are invaluable in revealing the common variants predisposing to complex human diseases. Yet, until now, the large volumes of data generated from such analyses have not been explored extensively enough to identify the molecular and functional framework hosting the susceptibility genes.

**Methodology/Principal Findings:**

We investigated the relationships among five neurodegenerative and/or autoimmune complex human diseases (Parkinson's disease-Park, Alzheimer's disease-Alz, multiple sclerosis-MS, rheumatoid arthritis-RA and Type 1 diabetes-T1D) by characterising the interactomes linked to their gwas-genes. An initial study on the MS interactome indicated that several genes predisposing to the other autoimmune or neurodegenerative disorders may come into contact with it, suggesting that susceptibility to distinct diseases may converge towards common molecular and biological networks. In order to test this hypothesis, we performed pathway enrichment analyses on each disease interactome independently. Several issues related to immune function and growth factor signalling pathways appeared in all autoimmune diseases, and, surprisingly, in Alzheimer's disease. Furthermore, the paired analyses of disease interactomes revealed significant molecular and functional relatedness among autoimmune diseases, and, unexpectedly, between T1D and Alz.

**Conclusions/Significance:**

The systems biology approach highlighted several known pathogenic processes, indicating that changes in these functions might be driven or sustained by the framework linked to genetic susceptibility. Moreover, the comparative analyses among the five genetic interactomes revealed unexpected genetic relationships, which await further biological validation. Overall, this study outlines the potential of systems biology to uncover links between genetics and pathogenesis of complex human disorders.

## Introduction

The aim of genome-wide association studies (gwas) is to discover the common genetic variants associated with susceptibility to complex diseases. In a typical experiment, hundreds of thousands of markers are tested simultaneously in cases and controls, and the allelic frequencies of each marker in the two groups are compared, so that the contribution of single genes to the disease is quantified. However, complex diseases do not originate from changes in single genes, but from the interactions between several genetic and environmental factors. Therefore, the analysis of isolated genes is not overly informative about the biological processes underlying disease and offers limited rationale for the development of novel therapies. Theoretically, reconstruction of the molecular interaction networks linked to gwas-genes by systems biology approaches may help to elucidate the functional consequences related to each susceptibility allele and the combined effects of more genetic variants. The few reports available to date on this issue demonstrated that this approach can identify previously unseen relationships among human diseases at molecular level [1], [2].

In this study, we elaborated the genetic interactomes relative to five complex human diseases. Our lab has strong interest in multiple sclerosis (MS), a chronic disorder of the central nervous system presumably of autoimmune etiology, characterized by inflammation of the white matter, demyelination and neurodegeneration [3]. Initially, we reconstructed the MS interactome and searched for interactions with genes predisposing to either neurodegenerative (Parkinson's disease Park, Alzheimer's disease Alz) or autoimmune (Type 1 diabetes T1D, Rheumatoid Arthritis RA) disorders. Then, disease interactomes of all five disorders were analyzed at the functional level by independent pathway enrichment studies. Finally, paired comparisons elucidated relatedness among the diseases. Interestingly, the shift from single genes to molecular frameworks via system biology unraveled novel functional relationships among the five complex diseases.

## Results

### Link between the MS interactome and the genes predisposing to other neurodegenerative or autoimmune diseases

**Figure 1:**
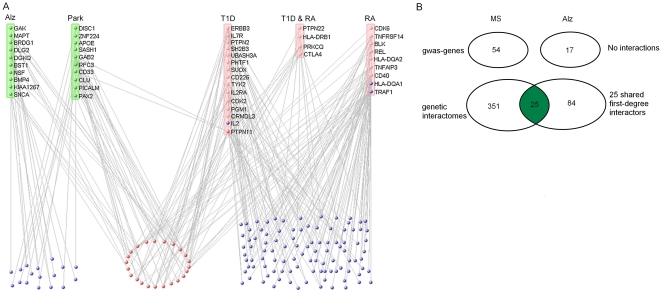
Relationship between MS interactome and gwas genes relative to other diseases. A. MS interactors shared by genes predisposing to neurodegenerative (green) and/or autoimmune (pink) diseases. Blue nodes indicate the MS interactors contacted by either neurodegenerative or autoimmune susceptibility genes. Red nodes indicate the MS interactors contacted by both neurodegenerative and autoimmune susceptibility genes. Note that four MS interactors are present among the T1D or RA gwas-genes. B. Comparison between MS and Alz at gwas-gene and genetic interactome levels. Despite the absence of a direct interaction at gwas level, shared molecular networks appear in the genetic interactomes (green section).

Initially we identified the susceptibility genes that were linked to MS and to other neurodegenerative (Park, Alz) or autoimmune (T1D, RA) disorders. We utilized results from 39 published genome-wide association studies on these five human diseases available in the GWAS catalog [4] and found genetic mutations in 179 genes passing the statistical significance threshold of 10^−5^ (Table 1). There were 6 studies available for MS, Park and RA, 8 for T1D, and 13 for Alz (Table S1). Notably, the number of studies carried out for each disease did not seem to influence the total number of disease-associated genes. For instance, despite the highest number of genome-wide association studies performed for Alz, the total number of susceptibility genes reported was the lowest among the five diseases. The ratios between the number of the reported genes and the number of studies in each disease accounted for this observation (Table 1). Interestingly, MS displayed the highest number of susceptibility genes and the highest ratio, suggesting a greater genetic heterogeneity in MS than in the other four diseases. Then, as a first step towards the definition of the genetic network underlying these five diseases, we reconstructed the MS genetic interactome and checked possible links with the genes predisposing to the other neurodegenerative or autoimmune diseases. Using the VisANT tool we derived 376 first-degree interacting partners for the 54 MS gwas-genes. Among these MS interactors, 141 were connected with at least one among 17 T1D, 11 RA, and 10 Park or Alz gwas-genes (Figure 1A and Table S2). Notably, 4 MS interactors were RA (HLA-DQA1, TRAF1) or T1D (IL2, PTPN11) gwas-genes themselves. It was also evident that several gwas-genes could come into contact with two or more MS interactors (Figure 1A), and that 24 MS interactors were connected with both neurodegenerative and autoimmune genes (red nodes in Figure 1A), including the PTPN11 gene.

**Table 1:** Summary of gwas data.

	MS	Park	Alz	T1D	RA	Total
**No. of studies**	6	6	13	8	6	39
**Unique genes**	54	35	17	52	21	179
**Gene/study ratio**	9	5.83	1.30	6.50	3.50	4.58

Now, the analysis of disease relatedness with MS is more effective when considering the genetic interactome rather than direct interactions at the gwas-gene level. In fact, no interactions existed between gwas-genes predisposing to MS and Alz, for example, but there were several shared interactions within the genetic interactomes linked to (but not including) the respective gwas-genes (Figure 1B). Therefore, the introduction of first-degree interactors in the definition of a disease-related molecular framework may lead to the discovery of relatedness among distinct complex disorders.

### Molecular relatedness among autoimmune and neurodegenerative genetic interactomes

To perform a global comparative analysis among autoimmune and neurodegenerative genetic interactomes, we derived a list of interacting partners for each gwas-geneset (Table 2, Table S3). As expected, the number of interactors was higher in MS and T1D, due to the more abundant number of reported gwas-genes. However, when normalizing the number of interactors to the total number of gwas-genes in each disease, RA reported the highest ratio among the five diseases, indicating that at least some of the RA susceptibility genes were highly interactive. In contrast, Park displayed the lowest interactor/gene ratio, despite the discrete number of described gwas-genes.

**Table 2:** Genetic interactomes for neurodegenerative and/or autoimmune disorders based on VisANT database.

	Genes	Interactors	Interactor/gene ratio
**Park**	35	160	4.57
**Alz**	17	109	6.41
**T1D**	52	316	6.07
**RA**	21	249	11.85
**MS**	54	376	6.96

**Figure 2:**
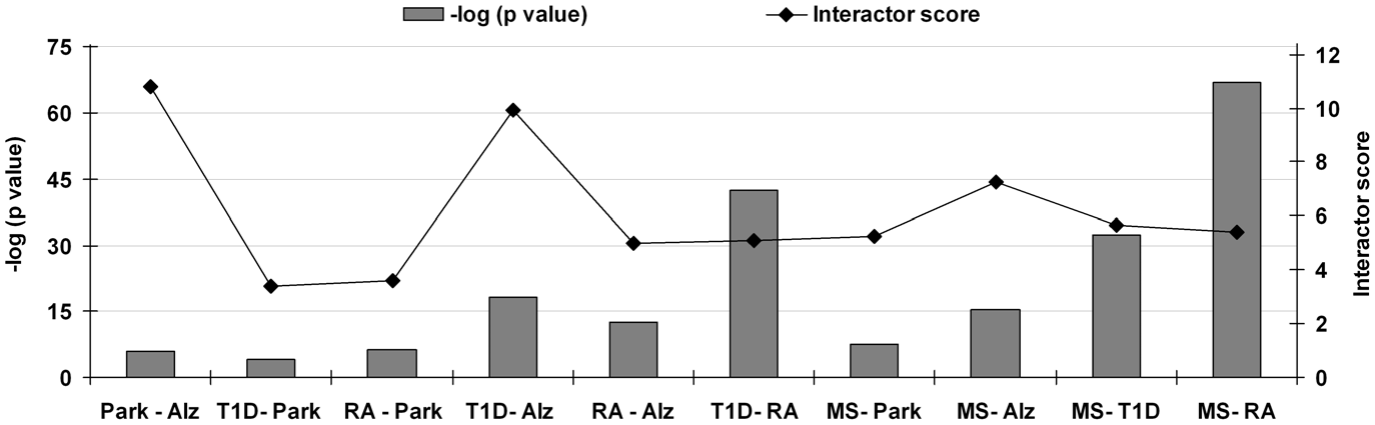
Molecular relatedness based on shared interactome. Statistical significance for each paired analysis is given.

Next, we examined the relationships among the five diseases by comparing the genetic interactomes in a pairwise fashion. The overlaps between interactomes are given in Table S4. Notably, higher concordances were found among the autoimmune diseases, with MS-RA, MS-T1D and T1D-RA having 84, 61 and 60 interactors shared respectively. Among the neurodegenerative diseases, the sharing of interactors was limited to 10 elements for Park-Alz, 20 for Park-MS and 25 for MS-Alz. Most surprisingly, 26 interactors were shared between the neurodegenerative Alz and the autoimmune T1D. The statistical significances for these observations were calculated using a hypergeometric test and are shown in Figure 2 (see bars) and Table S5. MS-RA had the lowest p-value (P = 1.02E-67), followed by T1D-RA (P = 4.42E-43) and MS-T1D (P = 3.09E-33). The T1D-Alz pair, with the p-value of 6.48E-19, was more significant than the MS-Alz pair (P = 5.21E-16). Comparatively higher p-values were found among T1D-Park (P = 1.18E-04), Alz-Park (P = 8.72E-07), RA-Park (P = 3.95E-07) and MS-Park (P = 3.36E-08), exhibiting the distant relatedness for all possible combinations with Park (Figure 2). Altogether, the results showed the close relatedness among autoimmune disorders and within Alz pairs.

The degree of relatedness may be due to the real biological properties of gwas-genes, i.e. the autoimmune gwas-genes participate in more biological pathways and processes than the neurodegenerative ones, and therefore the probability of abundant interactor sharing among autoimmune diseases is higher. However, we hypothesized that the observations could be partly biased by the difference in the number of studies carried out on distinct gwas-genes, consequently resulting in higher or lower interactor information. To critically assess this aspect, we considered that the average number of interactions per gene in the VisANT database was equal to 7.26 (93684 interactions for 12888 human genes). Consequently, we derived a *normalization factor* by normalizing each disease interaction ratio to the one in the VisANT database (Table S5). For example, the highest interaction ratio seen in RA resulted in a *normalization factor* equal to 1.88, signifying a nearly two fold increase in the interaction ratio compared to the database. Vice versa, Park normalization factor was 0.71, indicating that the interaction ratio was only 71% of that expected.

For paired analyses we multiplied the *normalization factors* relative to the two diseases (see materials and methods), and used the *paired normalization factor* to optimize the observed/expected ratio of interactor sharing. This resulted in an *interactor score* for each disease pair (Figure 2). So, if on the one hand the p-values relative to the inter-disease relatedness may reflect the shared interactome among diseases, on the other hand the interactor score might flatten or enhance some of these observations as it corrects for annotation bias. Therefore, both the p-values and the interactor scores have to be considered while interpreting the results. The interactor scores confirmed the close association among disease pairs in the autoimmune group (Figure 2). Surprisingly, the highest scores appeared in Alz-Park and T1D-Alz pairs. Therefore, even after eliminating the possible bias introduced by database annotation, the T1D-Alz pair maintained high association levels.

### Biological themes in autoimmune and/or neurodegenerative interactomes

**Figure 3:**
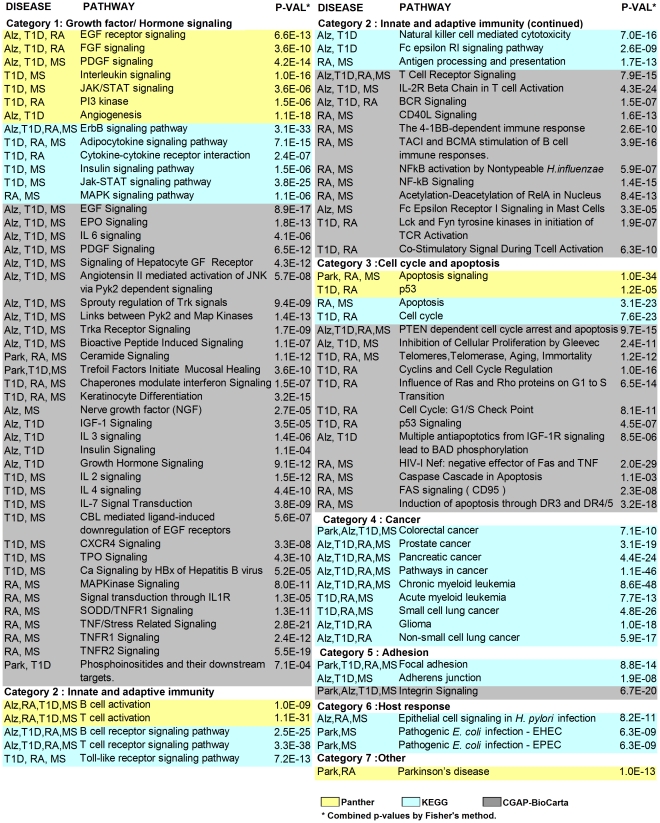
Pathways shared among diseases. Colors refer to the three distinct databases used for the pathway enrichment analysis. The combined p-values for pathway overlap among diseases are given.

In order to identify the biological themes embedded in each interactome, we used the ToppGene suite, an online tool for functional enrichment analysis. A Bonferroni corrected p-value of 0.05 was used to extract the significant biological pathways reported by three distinct databases (CGAP-BioCarta, KEGG and Panther). The highest number of pathways was reported in T1D followed by MS, RA, Alz and Park respectively (Table S6). Furthermore, we tabulated the shared pathways among diseases and grouped them according to the database in seven main categories: *Growth factor/Hormone signaling*, *Innate/Adaptive immunity*, *Cell cycle and apoptosis*, *Cancer*, *Adhesion*, *Host response* and *Other* (Figure 3). The combined statistical significance of the pathway enrichments is also listed in Figure 3. The majority of shared pathways were categorized in *Growth factor/Hormone signaling*, followed by *Innate/Adaptive immunity* and *Cell cycle and apoptosis*, while categories like *Cancer, Host response* and *Other* appeared in single databases.

In the *Growth factor/Hormone signaling* category, Panther indicated *EGF, FGF* and *PDGF signaling pathways* as shared by three diseases, Alz and T1D with MS or RA. The other pathways were shared exclusively by autoimmune diseases, with the exception of *Angiogenesis* that appeared both in T1D and Alz. In contrast to Panther, KEGG evidenced predominant pathway sharing among autoimmune disorders, except for the *ErbB signaling* present in Alz and in the three autoimmune diseases. CGAP-BioCarta exposed high concordances among Alz, T1D and MS. There were 10 pathways shared exclusively by these three diseases, among which *EGF signaling* and *PDGF signaling* were already reported by the Panther database. Many of the pathways common to these trios were related to tyrosine kinase signaling, such as *Trka receptor signaling* and *Sprouty regulation of tyrosine kinases*. Another remarkable observation was the sharing of the *NGF pathway* by MS and Alz. *IGF-1 signaling*, *IL3 signaling*, *Insulin signaling* and *Growth hormone signaling* pathways were shared exclusively by T1D and Alz. In addition, Park shared only three pathways with the autoimmune diseases, namely *Ceramide signaling, Trefoil factors initiate mucosal signaling* and *Phosphoinositides and their downstream targets*. Overall, pathways shared by the five diseases in the *Growth factor/Hormone signaling* category portrayed an undisputable association within the autoimmune group with the predominant presence of T1D. It also revealed that several biological themes related to tyrosine kinase signaling were shared among Alz, T1D and MS. Most unexpectedly, the analysis exposed numerous growth factor related pathways common to T1D and Alz.

The second category *Innate and adaptive immunity* contained 20 pathways derived from the three pathway databases. Although expected for autoimmune diseases, consistent sharing of immunity related pathways was also found in Alz. For example, pathways related to B and T cell activation appeared in all databases as shared by T1D, RA, MS and Alz. Furthermore, in the KEGG database Alz and T1D exclusively shared the *Fc epsilon RI signaling pathway* and *Natural killer cell mediated cytotoxicity* pathway, whereas CGAP-BioCarta emphasized predominant pathway sharing between MS and RA.

In the third category, *Cell cycle and apoptosis*, Panther and KEGG contributed with 2 pathways each, and Park shared the *Apoptosis signaling pathway* with RA and MS. Among the CGAP-BioCarta results, Alz shared 3 pathways with autoimmune diseases, while 4 pathways were shared exclusively by T1D-RA or RA-MS disease pairs.

The fourth category contained pathways derived from the KEGG database related to *Cancer*. It resulted that many genes appearing in autoimmune and/or neurodegenerative interactomes played a role in cancer related pathways.

In the fifth category (named *Adhesion*), the *Focal adhesion pathway* (KEGG) was common to Park and the autoimmune group and the *Integrin signaling pathway* (CGAP-BioCarta) was shared among neurodegenerative disorders and T1D. In addition, the *Adherens junction* pathway in KEGG was shared among Alz, T1D and MS.

The sixth category contained pathways related to *Host response*, which was reported exclusively by the KEGG database. Park and MS shared two pathways related to *E. coli* infection, and the autoimmune diseases shared the *Epithelial cell signaling in Helicobacter pylori infection* pathway. Lastly, the category *Other* contained the *Parkinson's pathway* reported by Panther, which was common to Park and RA.

Overall, pathway analysis revealed predominant sharing of functions among autoimmune diseases. Moreover, many of these pathways appeared also in Alz, which was associated with T1D in most cases.

### Quantitative analysis of functional relatedness among neurodegenerative and autoimmune interactomes

**Figure 4:**
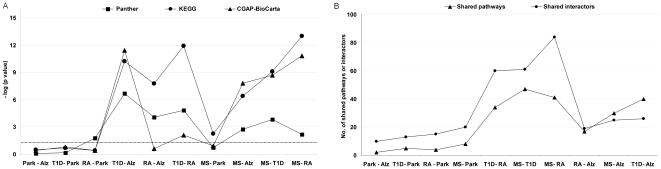
Molecular vs. functional relatedness. A. Functional relatedness based on shared pathways. Statistical significance for each paired analysis is given as relative to the database. The dotted line indicates the p-value threshold of 0.05. B. Shared pathways and interactors among disease pairs.

We tabulated the shared pathways for all disease pairs (Table S7) and quantified the significance of observed pathway overlaps by performing a hypergeometric test for each database separately (Figure 4A). According to Panther, the most significant pathway overlap was in the T1D-Alz pair (P = 2.10E-07), followed by the T1D-RA (P = 1.48E-05) and the RA-Alz (P = 8.69E-05). The MS-Park pair displayed no significant pathway overlap (P = 0.184). Among the KEGG results, the most highly significant pathway overlap was between MS and RA (P = 1.03E-13), followed by T1D-RA (P = 51.22E-12), T1D-Alz (P = 5.91E-11), MS-T1D (P = 8.58E-10) etc. Yet, the analysis revealed that pathway overlaps with Park could be due to chance, except for the MS-Park pair (P = 5.44E-03). Finally, the CGAP-BioCarta analysis recognized T1D-Alz as the most significant association (P = 3.71E-12), followed by MS-RA (P = 1.60E-11), MS-T1D (P = 2.16E-09), MS-Alz (P = 1.60E-08) etc. To summarize, the p-values based on KEGG and CGAP-BioCarta databases were more significant among the disease pairs in the autoimmune group, especially MS-RA. Moreover, on the basis of consistent statistical results in all the three databases, the T1D-Alz pair can be considered as the most significant disease pair in the context of shared pathways.

Theoretically, pathway sharing was partly anticipated as consistent interactor sharing was present in several disease pairs. Clearly, the more interactors were shared, the more common pathways were expected. We critically evaluated this issue and measured the Pearson's correlation between the interactors and the pathways (as reported by the three databases together) shared among the disease pairs. Consistently, we observed that the number of shared pathways was proportional to the number of shared interactors in most cases (Figure 4B). However, the Alz pairs with autoimmune diseases were not following this trend. The overall Pearson's correlation coefficient was 0.79, which increased remarkably to 0.95 when Alz-autoimmune disease pairs were excluded. These observations demonstrate that the number of shared biological pathways among Alz and autoimmune diseases is higher than expected on the basis of interactor sharing.

**Figure 5:**
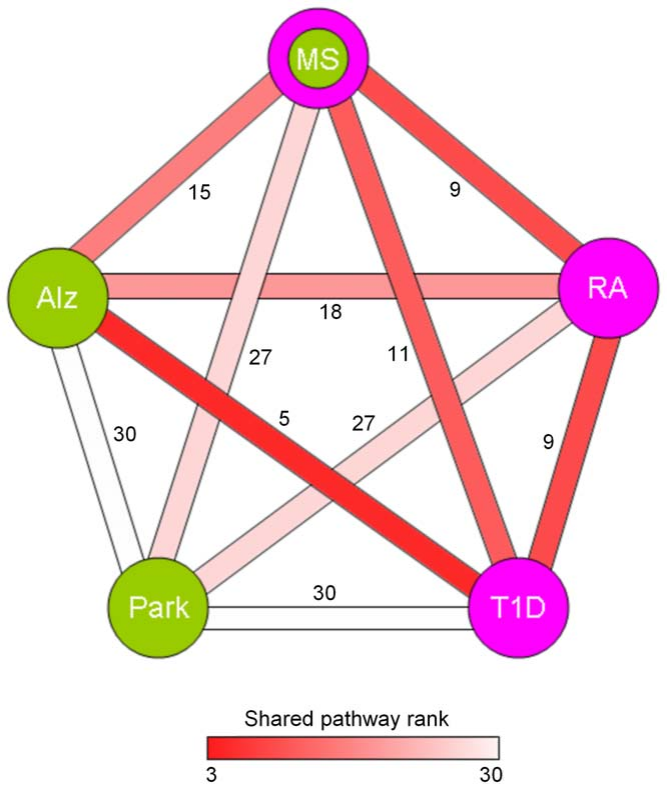
Overall disease relatedness based on shared pathways in the Panther, KEGG and CGAP-BioCarta databases. Green nodes indicate the neurodegenerative disorders, whereas pink nodes highlight the autoimmune diseases. The color of the edges connecting the nodes reflects the shared pathway rank ranging from 3 (highest relatedness) to 30 (lowest relatedness).

Finally, we ranked the associations among diseases based on the statistical significance of shared pathways in each database (Table S6). Scores starting from 1 were assigned from the highest significant association to the lowest. Those with insignificant p-values were assigned the rank 10. Then, we derived the cumulative ranks for each disease pair by summing their ranks in the three databases. As expected, T1D-Alz pair had the highest rank, followed by the autoimmune disease pairs MS-RA and T1D-RA, and then by MS-Alz and RA-Alz. All Park pairs scored very low. These results were depicted in Figure 5 displaying a network summary of relationships among the five diseases.

Altogether, the relationships among diseases identified at the level of molecular interactions were found to be sustained at the level of biological pathways, especially among autoimmune diseases. Surprisingly, the same approaches evidenced strong relatedness between Type 1 diabetes and Alzheimer's disease.

## Discussion

The reconstruction of the molecular framework hosting genetic variants associated with susceptibility to a complex human disorder has the potential to reveal the biological mechanisms underlying that disease and to highlight similarities with other diseases. Here we made a cross-disease comparison of autoimmune (T1D, RA) and neurodegenerative diseases (Park, Alz) starting from the point of view of MS, as it presents both autoimmune and neurodegenerative facets [3]. Indeed, several nodes in the MS interactome interacted with the susceptibility genes of the other four diseases, with a subset of MS interactors making contact with both autoimmune and neurodegenerative gwas-genes. These observations indicate that MS offers a framework that, to a certain extent, can be shared by other diseases. This is partly due to the fact that genetic susceptibility to autoimmune disorders may be driven by variations in some common genes [5], [6]. In this regard, two scenarios have been demonstrated. In the first case, the same variant may be involved in susceptibility to distinct diseases. For instance, genetic variation in the CLEC16A gene is associated with MS as well as T1D in the Sardinian population [7]. In the second case, distinct variants in the same gene predispose to distinct diseases, i.e. polymorphisms in the IL2RA gene linked to MS or T1D susceptibility are different [8], [9].

Previous studies have shown that molecular relatedness between distinct diseases may be found when searching for direct relationships among gwas-genes [10], [11] and that the analysis of first-degree interactors may also lead to similar observations[1], [2]. However, the advantage of the second approach compared to the first one has never been specifically addressed. Our study shows that the analysis of first-degree genetic interactomes may highlight shared molecular frameworks undetectable at the gwas-gene level. In fact, even when no interactions exist between gwas-genes, several shared interactions may be found when considering the genetic interactomes.

Furthermore, we elucidated the interactors linked to the gwas-genes in all five diseases and the pathway enriched in each disease-related interactome using the ToppGene suite. This tool contains a meta-database of annotated pathway databases such as Panther, KEGG, and CGAP-BioCarta. As the level of annotation and the number of genes related to a pathway may vary among databases [12], the outputs were not pooled but shown in relation to each database. Although a few previous studies revealed enriched biological pathways related to susceptibility genes in several complex human disorders, including MS [10], [13]–[15], no information was available on pathways emerging from the global genetic framework. In contrast, our study ascertained such issue in multiple sclerosis as well as in four additional autoimmune or neurodegenerative disorders. Independent pathway analyses demonstrated several commonalities among distinct genetic interactomes. Numerous immune related pathways were enriched in autoimmune interactomes. This result was expected as many of the susceptibility genes in RA, T1D and MS were immune related. Notably, the pathways *B-cell activation* and *T-cell activation* appeared in all autoimmune diseases. It is well known that both arms of adaptive immunity greatly contribute to autoimmunity. Our data suggest that the processes leading to alterations in immune tolerance may be caused or sustained by the genetic framework, and, hence, support the rationale for therapeutical approaches targeting T and B lymphocytes in autoimmune disorders. Surprisingly, the same pathways appeared in Alzheimer's disease. The role of adaptive immunity in Alz so far remains under-explored, however some studies suggest altered T cell phenotypes and responses in such patients (reviewed by [16]). Interestingly, regular use of anti-inflammatory drugs reduces the odds of developing Alz [17], [18]. Our observation that the Alz genetic framework may have an impact on immune function questions the classical distinction between inflammatory and non-inflammatory diseases and supports the hypothesis that, even though the primary insult is not inflammation but neurodegeneration, immunological pathways play a role in the etiopathogenesis of Alzheimer's disease [19].

Most of the enriched pathways appeared in the category *Growth factor/Hormone signaling* consistently among the three pathway sources. Panther and CGAP-BioCarta reported the EGF related pathway as enriched in Alz, T1D and RA or MS respectively, while KEGG highlighted the ErbB signaling pathway in all four diseases. EGF receptor belongs to the ErbB gene family. Interestingly, EGF is decreased in liquor from multiple sclerosis patients [20], while increased in the synovial fluid of RA patients, where it may regulate the inflammatory process [21]. Moreover, EGF promotes the release of amyloid precursor protein (APP) [22], indicating that it might support amyloidogenesis in Alz. Finally, downregulation of the EGF receptor signaling in pancreatic islets causes diabetes [23]. Our data suggest that the involvement of the EGF pathway in the pathogenesis of various complex human disorders may be genetically determined. The FGF signaling pathway appeared in two databases as enriched in T1D and Alz. Interestingly, biological evidence links alterations in this pathway to the two diseases, e.g. attenuation of FGF signaling in mouse beta cells leads to diabetes [24], and aFGF levels in liquor are increased in Alzheimer's patients [25]. Insulin related pathways were also enriched in T1D and Alz interactomes. Obviously, the major defect in T1D is insulin deficiency caused by the autoimmune attack against pancreatic β-cells. Accumulating evidences indicate that alterations in insulin signaling may contribute to Alz pathology (reviewed in [26]). Moreover, tau phosphorylation is increased in diabetic animals [27], and mice with combined APP overexpression and diabetes show exacerbated histological features of Alz [28]. Another interesting overlap is the angiogenesis pathway shared exclusively between Alz and T1D, especially if interpreted in relation to blood brain barrier dysfunction due to increased vascular permeability induced by hyperglycemia [29]. Further sharing of *Growth factor/Hormone signaling* pathways between Alz and T1D strongly supports the hypothesis of a genetic and functional link between these two disorders.

CGAP-BioCarta highlighted TNF related pathways both in RA and MS genetic interactomes. TNF is an inflammatory mediator clearly involved in the RA and MS pathology [30], [31]. Intriguingly, various biological compounds targeting TNF resulted effective in RA treatment [32], while detrimental in multiple sclerosis [33], indicating that TNF related pathways may play a dual role in autoimmune diseases.

The *Cell cycle and apoptosis* category was almost dedicated to autoimmune diseases. Such pathways regulate induction of immune tolerance and, indeed, changes in the balance between cell proliferation and death may lead to autoimmunity [34], [35]. The same processes may be altered in neoplastic cells. Cancer related pathways also consistently appeared enriched in the KEGG database. Overall, our data indicate that the genetic framework predisposing to complex human disorders may contribute to changes in these basic cellular functions.

The paired comparisons of genetic interactomes allowed measuring the degree of relatedness among the five disorders. Sharing of interactors and pathways was highly significant among the autoimmune group. This may be partly explained by the genetic overlap between autoimmune diseases [5], [6]. In fact, among all autoimmune gwas-genes analyzed in our study, HLA-DRB1 was associated with MS, T1D and RA, three genes (PTPN22, PRKCQ, CTLA4) were shared between T1D and RA, three (IL2RA, IL7R and CLEC16A) between MS and T1D, and one more (CD40) between MS and RA. However, analyses at the genetic network level also showed that several gwas-genes specific for single pathologies converged at the interactome level, meaning that, although the primary events may differ, the resulting functional cascades may come together and lead to alterations in the same pathways.

The most surprising observation was the strong correlation in the T1D-Alz pair in terms of shared interactome and functions. Moreover, this pair was found highly related by consistent performances in the three distinct pathway databases. Clinical and epidemiological data are available on associations between T1D and Alz. Type 1 (and Type 2) diabetic patients present deficits in numerous cognitive functions (reviewed in [36]) and diabetes is a risk factor for Alz [37]. In addition, biological evidence indicates that dysregulation of insulin metabolism may affect amyloid-β accumulation and degradation [38].

In conclusion, in this article we provided an unprecedented comparison among the genetic interactomes derived from genes predisposing to five complex human disorders. The shift in the network analysis from the gwas-genes to their first-degree interactors made the detection of shared molecules possible even when no interactions were present at the gwas-level. Furthermore, it revealed strong molecular and functional relatedness among autoimmune disorders. For example, the genetic interactomes pertaining to autoimmune diseases converged on numerous routes regarding immunity and growth factor signalling pathways. So, network generation and functional annotation highlighted several known pathogenic processes, indicating that changes in these functions might be driven or sustained by the framework linked to genetic susceptibility. Finally, the same tools underlined several inherent relationships among the five diseases at the level of genetic interactomes and biological pathways which went unnoticed until now. Type 1 diabetes and Alzheimer's disease were emblematic in this respect, as they appeared the most closely related disorders among all the disease pairs due to the extensive sharing of interactors and functions.

Overall, this study established that the reconstruction of the molecular framework hosting the genetic variants predisposing to complex human disorders can significantly contribute to our understanding of the biological functions linked to susceptibility genes. Many of these *in-silico* results highly correlate with the present experimental biology evidence, proving the reliability of systems biology tools. Furthermore, the study revealed unexpected genetic relationships, which await further biological validation.

## Materials and Methods

### Diseases and genetic association data

We collected genome wide association data related to five human diseases (T1D, RA, Park, Alz and MS) from the GWAS catalog (December 2009 version). The GWAS database is an online resource comprising of all published genome-wide association studies or meta-analyses of them, and provides information regarding the gene, SNP variations and their statistical significance in each study. We retrieved all the genes predisposing to each disease with the default critical p-value (10^−05^) of the database. The list of genes associated with each disease is summarized in Table 1 and the detailed records about each study such as sample size, journal name, authors, site of polymorphism etc. are summarized in the Table S1.

### Interactome analysis

To derive the list of interacting partners of the gwas-genes, we used the VisANT tool (December 2009 version) [39], a web-enabled program for construction, visualization, and analysis of molecular and higher order networks based on functional and physical relations of the genesets. Further, VisANT retrieves the interaction information from databases such as BIND, MIPS, Biogrid, HPRD and CAGT. The lists of interactors are given in Table S3. The statistical significances for the interactor overlap among disease pairs were calculated using a hypergeometric test. In order to compensate the eventual biases in database annotation, we calculated the *interactor score* through the normalization of the observed/expected ratio. In the VisANT database there were 93684 interactions among 12888 *Homo sapiens* genes, resulting in an average of 7.26 interactions per gene (VisANT interaction ratio). This ratio was used for normalization, as follows:

Disease interaction ratio  =  No. of interactions/No. of gwas-genes.

Normalization factor  =  Disease interaction ratio/VisANT interaction ratio.

Paired normalization factor  =  (Disease1 normalization factor) x (Disease2 normalization factor).

Observed  =  No. of interactors shared within a disease pair.

Expected  =  (No. of interactors in disease1/12888) x (No. of interactors in disease2).

Interactor score  =  (Observed/Expected) x (Paired normalization factor).

### Pathway enrichment study

We chose the ToppGene suite for determination of pathways enriched in the genetic interactomes. At the time of analysis, this tool maintained a meta-database of pathway information derived from KEGG (update: June 2009), Panther (update information not available), CGAP-BioCarta (update: August 2009). The analysis was performed with the stringent criteria of Bonferroni corrected p-value cut-off 0.05 in all cases. The combined p-values were calculated for the shared pathways among diseases using Fisher's method [40].

## Supporting Information

Table S1 Detailed records about the studies in the GWAS catalog used in our analyses.
(XLS)

Table S2 Overlap of MS genetic interactome with genes predisposing to other neurodegenerative or autoimmune diseases.
(XLS)

Table S3 List of the gwas genes and their interactors.
(XLS)

Table S4 Shared interactors in disease pairs.
(XLS)

Table S5 Statistical significances for interactor sharing and interactor scores.
(XLS)

Table S6 Statistical significances for pathway sharing and ranking of disease associations.
(XLS)

Table S7 List of shared pathways in disease pairs.
(XLS)
